# Acute effects of remote ischemic preconditioning on cutaneous microcirculation - a controlled prospective cohort study

**DOI:** 10.1186/1471-2482-11-32

**Published:** 2011-11-23

**Authors:** Robert Kraemer, Johan Lorenzen, Mohammad Kabbani, Christian Herold, Marc Busche, Peter M Vogt, Karsten Knobloch

**Affiliations:** 1 Plastic, Hand and Reconstructive Surgery, Hannover Medical School, Carl-Neuberg-Strasse 1, 30625 Hannover, Germany; 2 Department of Nephrology, Hannover Medical School, Carl-Neuberg-Strasse 1, 30625 Hannover, Germany

**Keywords:** Remote ischemic preconditioning, cutaneous microcirculation, free flap, soft tissue

## Abstract

**Background:**

Therapeutic strategies aiming to reduce ischemia/reperfusion injury by conditioning tissue tolerance against ischemia appear attractive not only from a scientific perspective, but also in clinics. Although previous studies indicate that remote ischemic intermittent preconditioning (RIPC) is a systemic phenomenon, only a few studies have focused on the elucidation of its mechanisms of action especially in the clinical setting. Therefore, the aim of this study is to evaluate the acute microcirculatory effects of remote ischemic preconditioning on a distinct cutaneous location at the lower extremity which is typically used as a harvesting site for free flap reconstructive surgery in a human in-vivo setting.

**Methods:**

Microcirculatory data of 27 healthy subjects (25 males, age 24 ± 4 years, BMI 23.3) were evaluated continuously at the anterolateral aspect of the left thigh during RIPC using combined Laser-Doppler and photospectrometry (Oxygen-to-see, Lea Medizintechnik, Germany). After baseline microcirculatory measurement, remote ischemia was induced using a tourniquet on the contralateral upper arm for three cycles of 5 min.

**Results:**

After RIPC, tissue oxygen saturation and capillary blood flow increased up to 29% and 35% during the third reperfusion phase versus baseline measurement, respectively (both p = 0.001). Postcapillary venous filling pressure decreased statistically significant by 16% during second reperfusion phase (p = 0.028).

**Conclusion:**

Remote intermittent ischemic preconditioning affects cutaneous tissue oxygen saturation, arterial capillary blood flow and postcapillary venous filling pressure at a remote cutaneous location of the lower extremity. To what extent remote preconditioning might ameliorate reperfusion injury in soft tissue trauma or free flap transplantation further clinical trials have to evaluate.

**Trial registration:**

ClinicalTrials.gov: NCT01235286

## Background

In several medical specialties, reduction of ischemia/reperfusion injury is a major topic. In trauma surgery, soft tissue defects after trauma due to accidents or due to operative trauma are quite common. In plastic and reconstructive surgery, on the other hand, soft tissue coverage is commonly performed using local or free flaps. Despite remarkable progress in surgical techniques and postoperative free flap monitoring devices over the last decades, free flap surgery is still associated with a significant morbidity. Current scientific literature reports a total loss rate of microsurgically transferred free flaps in one to five percent [1,2]. The main reasons are either persistent postoperative ischemia and hypoxia or transient ischemia associated with ischemia reperfusion injury [3]. Reducing or preventing soft tissue or free flap necrosis by conditioning tissue tolerance against ischemia is paramount in development of new treatment strategies. Current scientific literature demonstrates a possible induction of ischemic tolerance of several tissues by application of stressors including sublethal ischemia, hyperthermia, hypothermia, drugs and growth factors [4,5].

Applying brief periods of sublethal ischaemia to the target tissue with subsequent reperfusion is a simple method of ischemic tolerance induction, which is called ischemic preconditioning (IP). The aim of IP is to increase the resistance of the tissue against the subsequent deleterious ischemia reperfusion injury [4]. Nitric oxide (NO) as well as adenosine release has been shown to regulate endothelial function and increase blood flow in ischemic preconditioning settings [6]. However, direct IP produces trauma to major vessels and direct stress to the target organ.

Remote ischemic preconditioning (RIPC) is a further development of IP where ischemia followed by reperfusion of one organ is believed to protect remote organs either due to a systemic release of biochemical messengers in the circulation or activation of nerve pathways, also resulting in a secondary release of messengers that have a protective effect. This protects the target tissue without trauma to major vessels or direct stress to the target organ. In animal models, RIPC performed at the limb, gut, mesenteric, kidney or skeletal muscle reduced myocardial infarct size [7].

Furthermore, ischemic preconditioning has already been shown to be effective in flap surgery, demonstrated by an ameliorated survival of tissue in a porcine latissimus dorsi flap model [8].

A considerable number of experimental studies confirmed successful IP in flap surgery [9-11]. Since the introduction of IP to flap surgery, various procedures have been discussed such as the ideal time point of IP application, the duration of the IP cycle and the total number of cycles to apply.

Although the efficiency of IP has been demonstrated numerously in experimental in-vivo models, 'classical' IP has rarely been used in the clinical setting to target ischaemia- and reperfusion-associated complications [12].

A recent clinical trial among patients suffering from myocardial infarction demonstrated an increased myocardial salvage after prehospital remote ischaemic conditioning. To date, no scientific investigation has evaluated the acute microcirculatory effects of RIPC on cutaneous soft tissue in order to potentially attenuate the ischemia-reperfusion-injury during soft tissue traumatization or free flap transplantation in a perioperative clinical setting. Therefore, we hypothesized that intermittent remote ischemic preconditioning is able to immediately effect cutaneous microcirculation at a distinct cutaneous location at the lower extremity at a potential adipocutaneous free flap location

## Methods

The research was carried out in accordance with the Declaration of Helsinki (2000) of the World Medical Association and was approved by the Institutional Ethics Committee in October 2010. The study was registered at ClinicalTrials.gov with the identifier number NCT01235286.

Written consent was obtained from each study subject prior to commencement of the investigation.

The study is reported according to the STROBE-guidelines (Strengthening the Reporting of Observational Studies in Epidemiology) [13].

### Study design and setting

The study was designed as a prospective, controlled cohort study.

Data assessment was performed at the Department of Plastic, Hand and Reconstructive Surgery, Medical School Hannover, Germany between May and September 2010 using combined Laser-Doppler and photospectrometry (Oxygen-to-see, Lea Medizintechnik, Germany) which is regularly used for free flap microcirculatory monitoring at our department to determine postoperative free flap microcirculation. In order to determine changes of microcirculation resulting from remote ischemic preconditioning at a representative location for soft tissue traumatization and free flap transfer, we chose a distinct area for microcirculatory assessment at the anterolateral aspect of the thigh in the means of an ALT- (anterolateral thigh-) flap. A standardized location for microcirculatory assessment was determined on the left leg of each participant between the proximal and distal third of a drawn line between the anterior superior iliac spine and the lateral aspect of the Patella.

The healthy subjects had to rest before starting data assessment in a horizontal position for 15 minutes. The probe was taped on the left upper leg in a standardized manner after localizing the measuring point. A blood pressure cuff was applied on the contralateral upper arm. Baseline data was assessed over 5 minutes before starting remote ischemia. Three circles of a five minute ischemia were applied at the contralateral right upper arm at suprasystolic levels. Parameters of microcirculation were assessed continuously over time. Microcirculation during the reperfusion phase was ascertained over 10 minutes after first and second remote ischemia and 15 minutes after the third remote ischemia.

### Participants

#### Study population and recruitment

Eligibility criteria were healthy male and female subjects aged 18 to 35 years. Measurements were performed at the Department of Plastic, Hand and Reconstructive Surgery, Medical School Hannover, Germany. Healthy subjects were recruited from the Medical School Hannover.

Exclusion criteria were soft tissue inflammation or osteomyelitis, peripheral arterial occlusive disease, vasculitis, chronic kidney or liver disease, cardiac dysfunction, arterial hypotension and any type of vasoactive medication, i.e. ß-blockers, calcium channel blockers, nitroglycerin or equal.

The study population was a consecutive series of participants defined by the aforementioned selection criteria.

### Variables

#### Determination of vital parameters of microcirculation

The determination of hemoglobin and the principle of blood flow measurement are combined in the O2C system. The optical method for measuring both, blood flow by Laser-Doppler technique and hemoglobin oxygenation and hemoglobin concentration in tissue by spectrometric techniques, has been described in detail elsewhere [14]. The local oxygen supply parameters, blood flow, oxygen saturation of hemoglobin, and the relative postcapillary venous filling pressures were recorded by an optical fiber probe. The fiber probe incorporates both the laser Doppler method and the broadband light spectrometry technique. The probe we used assessed data in 2 and 8 millimeters depth of the free flap regarding:

• capillary blood flow [arbitrary units AU]

• tissue oxygen saturation [%]

• relative postcapillary venous filling pressure [AU]

### Bias

Structural measurement bias was avoided by standardisation of environmental factors during assessment of microcirculatory data, i.e. assessment by the same examiner, uniform subject's position during measurement, ambient light and temperature standardisation. Artefacts from microcirculatory measurements were tried to be avoided by fixation of the measurement probe on the same distinct location at the left upper leg of each subject.

The outcome variable selection bias was avoided by presenting all assessed microcirculatory variables in the paper that represent separate factors of microcirculation. Also, insignificant measures were presented. Literature retrieval biases were prevented by independent literature research through the National Library of Medicine. Funding bias was eliminated by disclosure of any financial support of any author of the paper.

### Statistical analysis

Repeated measures ANOVA and a Bonferroni-Holm-procedure as standard in multiple t-tests of statistical analysis were applied for comparison of baseline pre-ischemic vs. post-ischemic microcirculatory changes. A p-value less than 0.05 was considered to indicate statistical significance. The SPSS statistical software package 16.0 for Windows (SPSS Inc., Chicago, Ill, USA) was used for statistical analysis.

## Results

### Participants' descriptive data

A total number of 27 healthy subjects (25 males, 2 females) were enrolled in the evaluation of microcirculatory effects of remote ischemic preconditioning at the Department of Plastic, Hand and Reconstructive Surgery, Medical School Hannover, Germany.

Mean subject's age was 24 ± 4 years. Mean body-mass-index was 23.3 ± 3.0. All subjects were healthy non-smokers without any history of arterial hypertension, diabetes mellitus or vascular disease. Two thirds of the participants performed sports activities on a regular basis.

### Microcirculatory analysis

#### Capillary blood flow

**Figure 1:**
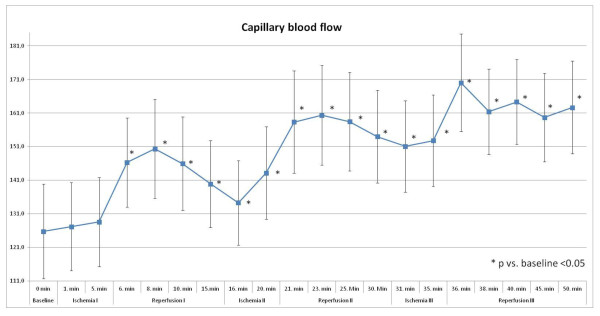
**Capillary blood flow [AU] with significant changes during ischemia- and reperfusion-phases**.

Cutaneous capillary blood flow increased by 16% during the first reperfusion phase at the anterolateral thigh, which reached statistical significance during the first minute after the first remote ischemia (126 ± 71 Arbitrary Units [AU] vs. 146 ± 67 AU; p = 0.001; Figure 1).

After ascending undulation of the microcirculatory blood flow during second and third remote reperfusion, the peak blood flow was reached straight after the third remote ischemia with an increase versus baseline measurement by 35% (126 ± 71 AU vs. 170 ± 73 AU; p = 0.001).

#### Postcapillary venous filling pressure

**Figure 2:**
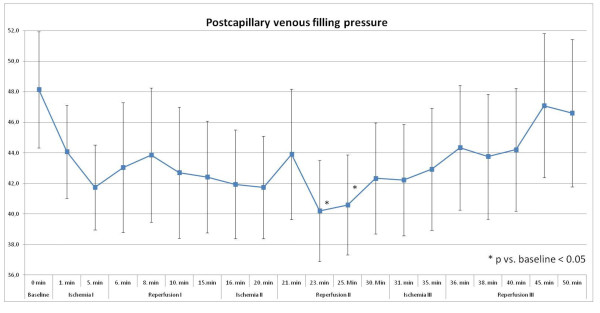
**Postcapillary venous filling pressure [AU] with significant changes during ischemia- and reperfusion-phases**.

Cutaneous postcapillary filling pressure at the anterolateral thigh decreased statistically significant 5 minutes after the second remote ischemia by 16% versus baseline (48 ± 19 AU vs. 41 ± 17 AU; p = 0.028; Figure 2).

#### Tissue oxygen saturation

**Figure 3:**
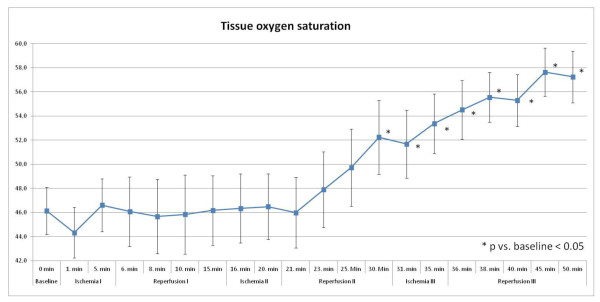
**Tissue oxygen saturation [%] with significant changes during ischemia- and reperfusion-phases**.

Tissue oxygen saturation assessed at the anterolateral thigh increased three minutes after remote ischemia reaching statistical significance eleven minutes after the second remote ischemia with an increase by 19% versus baseline measurement (46 ± 10% vs. 55 ± 12%; p = 0.021; Figure 3). The highest tissue oxygen saturation was detected 10 minutes after the third remote ischemia showing an increased oxygen saturation by 29% versus baseline measurement (46 ± 10% vs. 59 ± 8%; p = 0.001).

## Discussion

### Key results

We hypothesized that remote ischemic preconditioning (RIPC) is able to immediately affect cutaneous microcirculation at a potential fasciocutaneous free flap location at the anterolateral thigh (ALT-) region in a human in-vivo setting. This prospective controlled clinical study could demonstrate a significant increase of cutaneous oxygen saturation at the anterolateral thigh (ALT) skin territory, a significant increase of the cutaneous capillary blood flow as well as a statistical significant decrease of the postcapillary filling pressure as a marker of venous stasis during reperfusion phases immediately after RIPC. Therefore, our primary hypothesis was supported. These results should be discussed in detail.

In trauma and reconstructive surgery, tissue survival is paramount. In spite of tremendous progress in surgical techniques notably in reconstructive microsurgery, soft tissue still is endangered by both the lack of reoxygenation and the reoxygenation-associated inflammation due to reperfusion injury, especially in soft tissue trauma as well as in free flap failure [15]. In order to reduce the deleterious effects of ischemia and reperfusion injury, several methods of ischemia tolerance induction were developed including sublethal ischemia, hyperthermia, hypothermia, drugs and growth factors [4,5].

In plastic and reconstructive surgery, effects of ischemic preconditioning were also evaluated in free flap transplantation leading to a significant reduction of muscle flap necrosis supposedly by increasing the critical ischemia time of the free flap's soft tissue.

With the aim of reducing the number of additional procedures and the invasiveness of the individual preconditioning measures, remote ischemic preconditioning (RIPC) was introduced. The ameliorating effect of RIPC was attributed to regulation of endothelial protection, whereas other studies about the mechanisms of preconditioning effects on muscle and musculocutaneous flaps disclosed a decrease of capillary no-reflow, attenuation of arteriolar vasospasm and an increase of blood flow response [11,16-18]. Furthermore, if ischemic preconditioning is applied directly before critical soft tissue ischemia, it protects both against the leukocytic inflammatory reperfusion injury and against microcirculatory dysfunction. On the other hand, ischemic preconditioning performed 24 hours or more before critical soft tissue ischemia acts anti-inflammatory, but is not capable of improving the microcirculation [19]. The short-term microcirculatory assessment in our study was performed straight after remote ischemia to detect acute cutaneous microcirculatory effects of remote ischemic preconditioning. The intention of this study was not to evaluate the protective effects of RIPC on soft tissue in general over days after trauma or operation, but to detect whether RIPC has effects on cutaneous microcirculation and if so what kind of effects these might be. No other study before has demonstrated microcirculatory effects of RIPC on skin which should also be considered to be an important body organ that must be protected against ischemia in several settings.

Although it is generally accepted that IP increases flap survival by enhancing microvascular perfusion, the molecular and microcirculatory mechanisms are still not completely understood. The noninvasive application of a tourniquet at the hindlimb to induce IP was introduced by Kuentscher et al. and demonstrated to be as effective as the invasive clamping of the flap's pedicle itself, including both adipocutaneous and muscle flaps [20].

Recently, remote ischemic preconditioning demonstrated a modulation of hepatic microcirculation with consecutive reduction of the effects of ischemic reperfusion injury in an in-vivo animal model. RIPC in that setting significantly increased red blood cell velocity, sinusoidal flow and sinusoidal perfusion along with decreased neutrophil adhesion and cell death. For remote preconditioning the tourniquet was tightened around the limb until no flow was detected. The procedure involved 5 minutes of ischemia followed by 5 minutes of reperfusion. This was repeated four times [21]. Regarding myocardial perfusion, Hooele et al. could not find an effect of RIPC on coronary microvascular resistance or coronary flow in humans [22]. Nevertheless, Thielmann et al. found that myocardial injury after coronary artery bypass grafting was reduced by RIPC [23]. Zimmermann et al. demonstrated RIPC as protective from acute kidney injury in patients following cardiac surgery. In that study remote ischemic preconditioning was applied by an automated thigh tourniquet consisting of three 5-minute intervals of lower extremity ischemia separated by 5-minute intervals of reperfusion. Within 48 hours after surgery there was a significant both absolute and relative risk reduction due to preconditioning [24]. Another recent trial found no evidence that remote ischemic preconditioning provided protection of kidney function in children undergoing operation for complex congenital heart disease. Four cycles of five minutes RIPC were applied in that study [25].

The clinical and experimental data about RIPC in current literature remains confusing. An equal microcirculatory effect of RIPC on different kinds of tissue obviously is not mandatory demonstrated in several studies. A dose effect dependency can still not be included in RIPC because of the fact that there is still a non-equivalent effect apparent in different trials with identical dosages of RIPC. The main reason for this heterogeneous scientific data could be the indisputable heterogeneity of clinical studies concerning with RIPC, especially in terms of methodology and cohorts [26]. Thus, basic scientific research on RIPC is mandatory and must include investigations on healthy cohorts. For this reason, we included only young, healthy subjects in our study and focused our investigation on immediate and short-term effects of RIPC on a currently scientifically non-investigated body area.

Current scientific data demonstrate that three cycles of IP are significantly superior to one or two cycles, regardless of whether the cycles were 5 min or 10 min [27]. According to that current literature, remote ischemia in our study was applied at the contralateral upper arm including five minutes of ischemia with ten and finally fifteen minutes of reperfusion over three circles leading to an increase of cutaneous oxygen saturation at the anterolateral thigh skin territory, a significant increase of the capillary blood flow as well as a statistical significant decrease of the postcapillary filling pressure as a marker of venous stasis.

As our study elucidated the short term results of RIPC and documented the beneficial effect of the intermittent, repetitive component of RIPC on microcirculation, further studies should now focus on intermediate and long term effects of RIPC on cutaneous microcirculation. Another field of application of RIPC should also be part of further investigations calling muscular microcirculation as ischemia of muscle does not necessarily lead to muscle necrosis, but can lead to progressive microvascular dysfunction at an early stage with consecutive microcirculatory impairment as a vicious circle up to a compartment syndrome [28]. Therefore, an increase of cutaneous oxygen saturation and capillary blood flow as well as a decrease of venous stasis as immediate effects of RIPC, which we demonstrated in our study, might also be beneficial in reoxygenation-associated inflammation due to reperfusion injury in muscular trauma or surgery.

### Limitations

This study enrolled 27 healthy subjects for microcirculatory assessment of RIPC. In spite of the cohort size, statistical analysis found significant changes of microcirculation. Nevertheless, further studies have to evaluate the correlation of the microcirculatory parameters and RIPC in a clinical setting before and after extended soft tissue traumatization or elective free flap transplantation.

## Conclusion

Remote ischemic preconditioning effects cutaneous tissue oxygen saturation, arterial capillary blood flow and postcapillary venous filling pressure at a distinct location of the lower extremity. Although, current scientific literature indicates that remote preconditioning is a systemic phenomenon, only a few studies have focused on the elucidation of its mechanisms of action. To what extent early and late remote preconditioning might influence the reperfusion injury of free flap transplantation, further clinical trials have to evaluate both in the means of microcirculatory assessment and clinical assessment of soft tissue necrosis due to traumatization or partial or total flap loss in free flap transplantation as end points of these studies.

## Competing interests

The authors declare that they have no competing interests.

## Authors' contributions

RK, JL, KK, PMV conceived of the study, participated in its design and coordination and helped to draft the manuscript. MK carried out the data assessment. CH, MB participated in the design of the study, drafted the manuscript and performed the statistical analysis. All authors read and approved the final manuscript.

## Pre-publication history

The pre-publication history for this paper can be accessed here:

http://www.biomedcentral.com/1471-2482/11/32/prepub
